# Label matters: how labeling and diagnosis affect lay perception of people with depressive symptoms

**DOI:** 10.3389/fpsyt.2025.1612517

**Published:** 2025-09-02

**Authors:** Katarzyna Kulwicka, Jagoda Rusowicz, Agata Gasiorowska

**Affiliations:** Faculty of Psychology in Wroclaw, SWPS University, Wroclaw, Poland

**Keywords:** depression, labels, labeling effect, diagnosis, perception of people with depressive symptoms

## Abstract

We investigated how the label “depression” and information about a medical diagnosis influence perceptions of individuals with depressive symptoms as legitimately experiencing depression and a medical condition. In three experiments, participants read a description of a person meeting Diagnostic and Statistical Manual of Mental Disorders (DSM-5) criteria for major depressive episode and manipulated whether the label “depression” and the information about a professional medical diagnosis were included. Participants were more likely to perceive the person as having depression when both the label and diagnosis were present. However, paradoxically, when a diagnosis explicitly included the word “depression”, participants were less likely to view the symptoms as indicating a legitimate medical condition than when the diagnosis omitted the term. These effects were not moderated by participants’ own experience of depression. Gender effects emerged in Experiment 3: results replicated for male protagonists but differed for female protagonists, where label effects were independent of medical diagnosis information. Finally, a meta-analysis across the three experiments supported our hypothesis that the label “depression” weakened the effect of the medical diagnosis. Moreover, participants attributed a higher degree of legitimacy to a medical condition when the diagnosis was provided by a doctor, but only when this diagnosis did not include the label “depression”.

## Introduction

Research on labels in the context of mental health and illness has a long history. The classical labeling theory of mental illness (1), popular in the 1970s and 1980s, initially focused on the identity of individuals diagnosed with a mental disorder. It was then expanded to cover the social consequences of psychiatric diagnoses (2), leading to the extensive research on the labeling effect and its impact on social response to people with mental illness (3, 4). Although the academic community has extensively studied this topic over the years, the understanding of how psychiatric labeling affects public perception and understanding of mental disorders is still limited. Our paper addresses this gap by investigating how the label “depression” influences lay people’s perception of this disorder. Specifically, we examined how this particular label and information about the medical diagnosis of depression influence public perceptions of people with depressive symptoms as legitimately experiencing depression and a medical condition.

According to the labeling theory of mental illness (1), labeling a person as “mentally ill” leads to two main outcomes. First, other people react to the labeled person according to the prevailing social concept of mental illness. Second, the labeled person adopts the role of “mentally ill” and develops a relatively stable identity around this specific role. This approach has been widely criticized for underestimating other identity-related consequences of the labeling process (5, 6). The result of the massive criticism was the development of the so-called modified labeling theory of mental illness (7), which, in contrast to the classical theory, focuses on the assumption that psychiatric labeling, defined as the use of psychiatric diagnoses when referring to or describing a person, has a profound negative impact on the lives of individuals diagnosed with mental disorders and leads to stigmatizing attitudes and the desire for greater social distance toward them (e.g., 8–10). In the classical study on the effect of labeling on the attribution of traits, people labeled as “psychiatric patients” were perceived as less sympathetic, less responsible, and less clear-thinking than people labeled as “medical patients” (11). The results of another study demonstrated that the additional single piece of information that someone is diagnosed with a “psychiatric condition” increased the declared social distance toward that person (12). This effect was also observed in medical students following their participation in a psychiatry course (13). Another study showed that participants who read a short description of a person with the additional information that this person had been diagnosed with “schizophrenia” perceived this person as more aggressive and less trustworthy and felt more fear of this person compared with participants who read the same description but without the label (14). Furthermore, labeling a person as “depressed”, compared to when the label was not provided, led to more negative reactions to that person’s comments (15) and lower levels of trust demonstrated toward those who are labeled (16). The power of the label on the perception of others was demonstrated in a recent study (17), where stigmatizing attitudes and desire to social distance toward a labeled person persisted even when the label was retracted.

Despite the considerable attention that the academic community has devoted over the decades to the question of how diagnostic labeling affects social responses to people diagnosed with mental disorders (3), little is known about how psychiatric labeling affects lay people’s perception and understanding of mental disorders. In one of the classical studies on the effect of labeling on the ascribed psychiatric diagnosis (18), psychiatrists and abnormal psychology students were asked to watch videotaped standardized psychiatric interviews with a mental health hospital patient and a paid participant. Some participants were told that the two interviews were conducted with a psychiatric patient, while others were told that both interviews were conducted with a student. Participants then briefly described the interviewee and rated their current level of illness or impairment. The term “mental patient” resulted in the students—but not the psychiatrists—indicating a higher level of diagnosed mental illness if the interviewee was a mentally ill person. If the respondent was a paid participant, the label significantly influenced both the students’ and professionals’ assessments.

Similar results were obtained in a study on the effect of labeling on the perceived “psychological incapacitation” of students (19). In this experiment, teachers were presented with the description of a hypothetical student together with information about the diagnostic labels, behavioral description of the student, or diagnostic label along with the behavioral descriptions. Perceived “psychological incapacitation” was significantly higher when a label was present than when it was not, regardless of whether behavioral descriptions were provided.

In another classic experiment, university students were presented with two recordings of a conversation between four people and were asked to evaluate one of them (20). Depending on the experimental condition, participants rated a person who was acting either casually or in a way that could be interpreted as disturbed. In addition, participants were informed that this person was described as “severely neurotic” by professionals, or they did not receive such information. The results of this experiment confirmed the labeling effect: participants attributed higher levels of psychological disorder to the protagonist when the label was present than when the label was not mentioned, suggesting that the label may affect not only the perception of the labeled person but also the perception of the severity of symptoms or mental health condition in general.

A recent study by Altmann et al. (21) demonstrated that the use of the “major depressive disorder” label increased the participants’ perception that people who experience minor problems require professional treatment and also increased the perceived persistency of the condition compared to “general anxiety disorder” and “bipolar disorder” labels. This is a rare example of studies investigating the effect of the label not only on the perception and attitudes toward a labeled person but also on the lay perception of mental health conditions.

Although in the public discourse the label “depression” is used as a term for various conditions—from everyday low mood to various psychiatric disorders (22, 23)—it carries specific clinical implications. A recent large corpus study of historical semantic change demonstrated the tendency to pathologize the concept of “depression”. The term is increasingly located in semantic contexts involving symptoms, disorder, and diagnosis alongside “anxiety” (24). Despite this pathologization trend, “depressive neurosis” remains one of the disorders of the least prestige as perceived by medical professionals (25). This, in turn, may result in underestimating the seriousness of the condition (26, 27).

Given that depression is the leading cause of disability worldwide and is one of the most common mental disorders globally (28, 29), we believe that investigating how the label “depression” itself affects lay recognition of depressive symptoms and how it influences the attribution of depressive symptoms as a mental disorder and medical condition is especially important.

## Overview of the studies

In three experiments, we presented participants with a vignette of a person experiencing symptoms of depression according to diagnostic criteria and manipulated whether this description was accompanied by 1) the label “depression” and 2) information about the medical diagnosis. We hypothesized that a protagonist would be perceived as having depression to a greater extent when the label “depression” is provided than when it is not, and that this effect would be stronger when accompanied by information about the medical diagnosis but weaker when there is no information about the medical diagnosis. We also investigated whether the label and information about medical diagnosis would affect the perception of the protagonist as having a medical condition.

In Experiment 1 (*N* = 684), we found that participants perceived the protagonist as having depression to a greater extent when the label was accompanied by information that depression had been diagnosed by a doctor, but not when there was no information about the diagnosis. The use of the label “depression” reduced the perception of the protagonist as experiencing a medical condition, even when the disorder was diagnosed by a doctor. In preregistered Experiment 2 (*N* = 1,526), we replicated these findings and determined that they were not moderated by participants’ own experience of depression. In Experiment 3 (*N* = 1,554), we investigated whether the results differed by gender of the described person (Margaret vs. John). We replicated our results for the male protagonist only. For the female protagonists, the effect of the label was independent of the effect of the medical diagnosis. A meta-analysis on Experiments 1–3 supported our hypothesis that the label “depression” weakened the effect of the medical diagnosis. Specifically, participants attributed a greater degree of legitimacy to the medical condition when the diagnosis was provided by a doctor, but only when this diagnosis did not include the label “depression”.

## Experiment 1

In this experiment, we provided participants with the description of the protagonist who experiences symptoms of a major depressive episode according to DSM-5 (30), yet formulated in everyday language, not in the so-called “voice of medicine” (31). Moreover, we manipulated whether this description was accompanied by the label “depression” and by information that a doctor diagnosed the protagonist’s condition. We examined whether these two factors would influence participants’ perception of how probable it is that the described person is experiencing depression and is experiencing a medical condition. We hypothesized that (H1) participants in the “depression” label condition would assess the probability that the person described in the vignette is experiencing depression as higher than participants in the no-label condition and that (H2) this effect would be stronger for participants in the medical diagnosis condition compared to the no diagnosis condition. Moreover, we expected that (H3) participants in the medical diagnosis condition would assess the probability that the person described in the vignette is experiencing a medical condition as higher than participants in the no diagnosis condition. As perceptions of the person as having depression and experiencing a medical condition were correlated, we controlled for the other dimension of perception in the analysis to obtain the effects specific to our experimental manipulations on the perception of depression and perception of actual medical condition.

### Materials and methods

We estimated the sample size using an *a prioria priori* power analysis with G*Power (32). Based on the previous studies on labeling effect (14), we assumed an effect of Cohen’s *d* = 0.3, an expected power 1 − β = .80, and a significance level α = .05. Therefore, we aimed to invite 176 participants for each of the four experimental conditions in a 2 (label vs. no label) × 2 (diagnosis vs. no diagnosis) between-subjects experimental design.

We recruited *N* = 704 U.S. Prolific Academic users to participate in this study for £0.50. We excluded 20 participants based on failed attention checks (“Enter today’s date in the day-month-year format” and “Please answer ‘Definitely willing’ in this question”). The final sample included *N* = 684 participants (338 women, 341 men, and 5 with no information) aged 18–85 years (*M* = 35.25, *SD* = 12.91).

After providing informed consent and demographic data, participants were assigned to one of the four conditions in a 2 (label vs. no label) × 2 (information on the medical diagnosis vs. no information on diagnosis) between-subjects design. They were asked to read a short description of a person with different final passages, depending on the condition:

“Imagine that some time ago, you met at a party someone named Alex. You found Alex interesting, and you had a good time talking to each other. You found out that Alex has a full-time job that she likes and is in a happy long-term relationship. A few weeks later, you called Alex to invite them for lunch. Alex kindly refused, saying that she had recently been in a worse mood than usual. She also mentioned that she doesn’t sleep well and is tired for most of the day. Later she also told you that somehow, she is not so satisfied with her job anymore, even if nothing at work had changed. She added that she has problems with concentration and cannot even read books, which used to be her biggest hobby. Even worse, she claimed that her relationship was not so satisfying anymore. She noticed that she is constantly irritated with her partner, feeling guilty about it.”

The final passage was as follows: in the control condition (*n* = 176), “Alex wondered what was going on with her”; in the “label” condition (*n* = 169), “Alex wondered whether she might have depression”; in the “medical diagnosis” condition (*n* = 169), “Alex went to see a doctor, and the doctor diagnosed that the way she feels is due to the medical condition”; and in the “label + medical diagnosis” condition (*n* = 170), “Alex went to see a doctor, and the doctor diagnosed that the way she feels is due to depression”.

Then, participants were asked to answer two questions— “In your opinion, how probable it is that Alex might have depression?” (*M* = 78.06, *SD* = 18.72) and “In your opinion, how probable it is that Alex might have some medical condition?” (*M* = 69.48, *SD* = 21.61)— using a scale from 0 = “Very improbable” to 100 = “Very probable”. These questions were the two dependent variables.

### Analytical approach

We conducted two analyses of covariance (ANCOVAs) in the 2 (label vs. no label) × 2 (information on the medical diagnosis vs. no information on diagnosis) factorial design. The first ANCOVA examined the perception of Alex as having depression as the dependent variable, with the perception of Alex as having a medical condition as a covariate. The second ANCOVA examined the perception of Alex as having a medical condition as the dependent variable, with the perception of Alex as having depression as a covariate.

### Results

**Figure 1:**
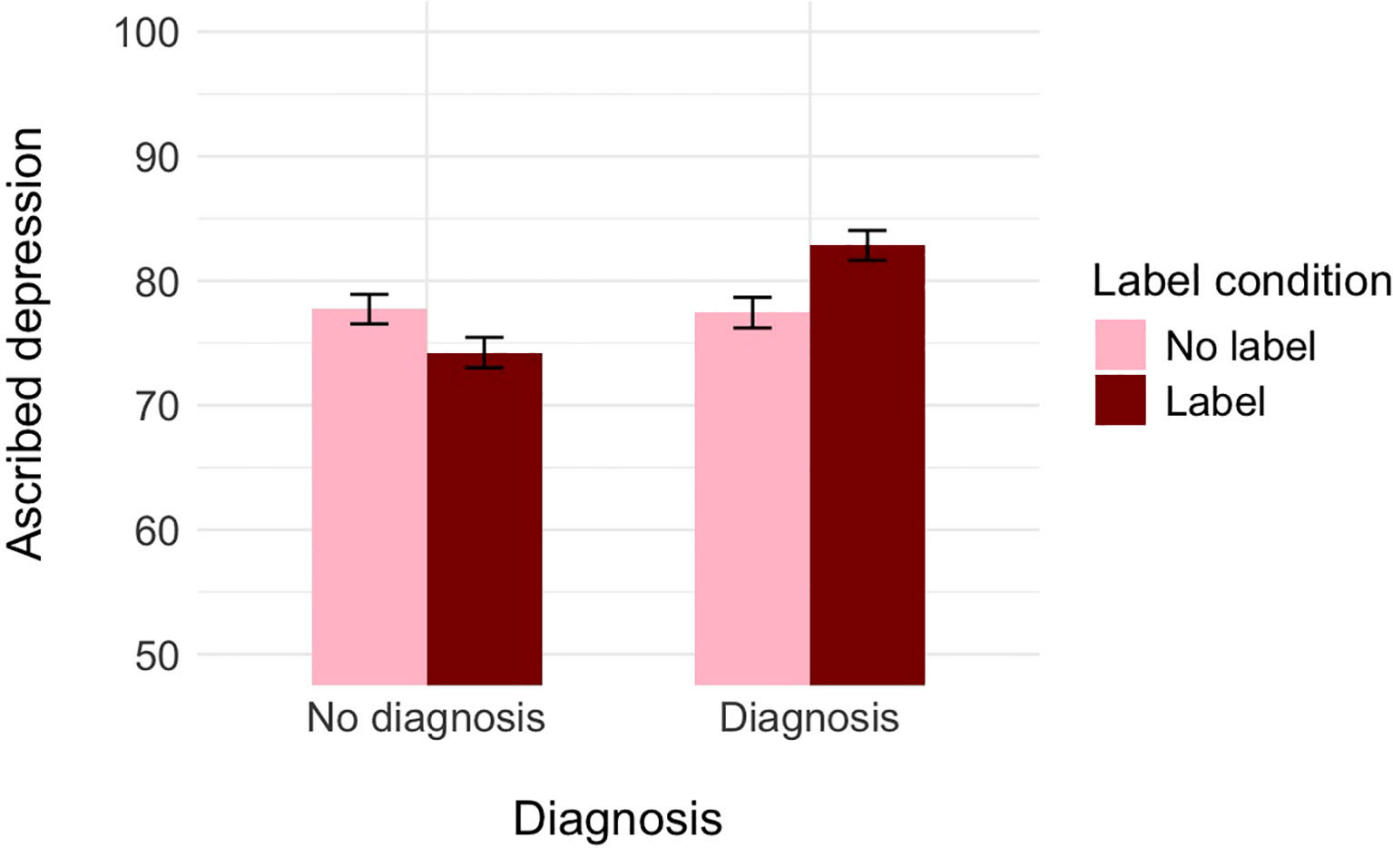
The effect of label and medical diagnosis on ascribed depression, controlling for the ascribed medical condition (Experiment 1).

In the first analysis, the associations between the two dimensions of perception were significant. The effect of diagnosis remained significant, the same as the interaction between the two factors, while the effect of label was not significant (see **Table 1** and **Figure 1**).

**Table 1:** Analysis of covariance for the effect of label and medical diagnosis on ascribed depression, controlling for ascribed medical condition (Experiment 1).

Predictor	*F* (1, 679)	*p*	η^2^_p_
Ascribed medical condition	184.93	<.001	.214
Diagnosis	11.4	<.001	.017
Label	0.62	.432	.001
Diagnosis × Label	13.75	<.001	.020

A further decomposition of the two-way interaction with the Bonferroni correction revealed that, controlling for the perception of the protagonist as having a medical condition, participants who did not receive the information about the diagnosis assessed the probability of Alex as having depression as similar independently of whether the label was provided (*M* = 74.22, *SE* = 1.18) or not (*M* = 77.72, *SE* = 1.22), *t*(679) = 2.07, *p* = .232, Cohen’s *d* = 0.22. In turn, participants who received the information about the diagnosis assessed the probability of Alex as having depression as higher when the label was provided (*M* = 82.84, *SE* = 1.20) than when it was not (*M* = 77.44, *SE* = 1.23), *t*(679) = −3.14, *p* = .010, Cohen’s *d* = −0.34. Alternative decomposition of the interaction revealed that within the no-label condition, participants who did not receive the information about the diagnosis assessed the probability of Alex as having depression as lower (*M* = 76.26, *SE* = 1.43) than those who received the information about the diagnosis (*M* = 81.07, *SE* = 1.26), *t*(684) = −2.53, *p* = .070, Cohen’s *d* = −0.27. In turn, within the label condition, participants who did not receive the information about the diagnosis assessed the probability of Alex as having depression as lower (*M* = 71.64, *SE* = 1.40) than those participants who received the information about both the label and the diagnosis (*M* = 83.33, *SE* = 1.26), *t*(684) = −6.10, *p* <.001, Cohen’s *d* = −0.66.

**Figure 2:**
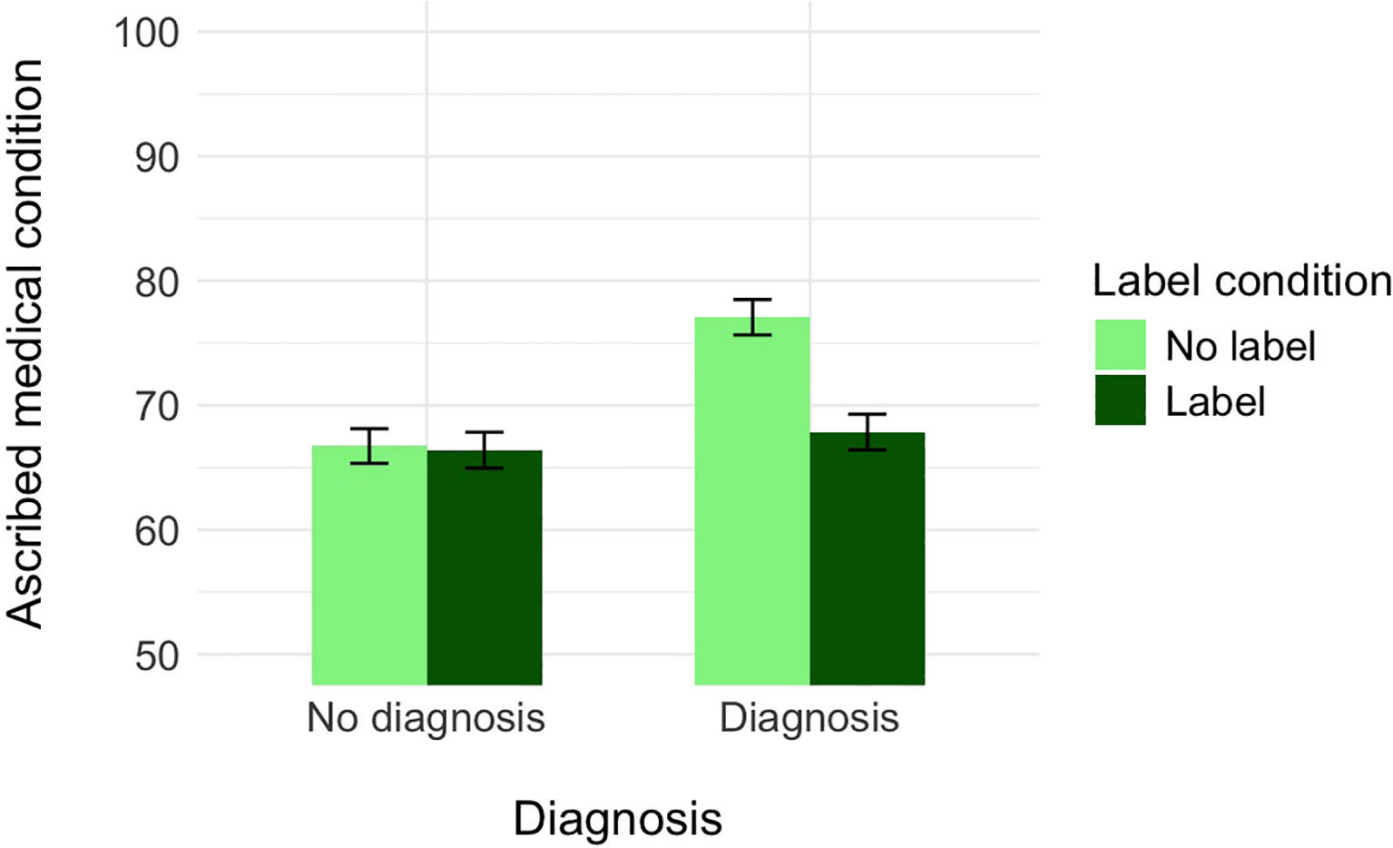
The effect of label and medical diagnosis on ascribed medical condition, controlling for the ascribed depression (Experiment 1).

In the second analysis, the association between the two dimensions of perception was significant. As expected, the effect of diagnosis was significant. Additionally, we found a significant effect of label and a significant interaction between label and diagnosis (see **Table 2** and **Figure 2**).

**Table 2:** The effect of label and medical diagnosis on ascribed medical condition, controlling for ascribed depression (Experiment 1).

Predictor	*F* (1, 679)	*p*	η^2p^
Ascribed depression	184.93	<.001	.214
Diagnosis	16.57	<.001	.024
Label	11.44	<.001	.017
Diagnosis × Label	9.82	.002	.014

A further decomposition of this interaction with the Bonferroni correction revealed that, controlling for the perception of the protagonist as having depression, participants who did not receive the information about the diagnosis assessed the probability of Alex as having a medical condition as similar independently of whether the label was provided (*M* = 66.39, *SE* = 1.44) or not (*M* = 66.72, *SE* = 1.39), *t*(679) = 0.17, *p* = .999, Cohen’s *d* = 0.02. In turn, participants who received the information about the diagnosis assessed the probability of Alex as having a medical condition as higher when the label was not provided (*M* = 77.07, *SE* = 1.43) than when it was (*M* = 76.85, *SE* = 1.43), *t*(679) = 4.59, *p* <.001, Cohen’s *d* = 0.50. Alternative decomposition of the interaction revealed that within the no-label condition, participants who did not receive the information about the diagnosis assessed the probability of Alex as having a medical condition as lower (*M* = 66.72, *SE* = 1.39) than those who received the information about the diagnosis (*M* = 77.07, *SE* = 1.43), *t*(684) = −5.18, *p* <.001, Cohen’s *d* = −0.56. In turn, within the label condition, participants who did not receive the information about the diagnosis assessed the probability of Alex as having a medical condition (*M* = 66.39, *SE* = 1.44) as similar to participants who received the information about both the label and the diagnosis (*M* = 67.85, *SE* = 1.43), *t*(684) = −0.71, *p* = .999, Cohen’s *d* = −0.08.

In sum, we found that the effect of the label on the perception that a protagonist has depression is significant when a medical diagnosis accompanies it, but not when there is no diagnosis. These results provide initial evidence that using the word “depression” does not legitimize the condition in the perception of lay people, and to provide such legitimization, one must be diagnosed by a doctor. Moreover, we also found that when a person is diagnosed by a doctor, lay people perceive the condition as less legitimate when the label “depression” appears than when the label is not indicated. In fact, when the label was mentioned along with the information about medical diagnosis, the perceived legitimacy of the condition was similar to the one without any diagnosis— for lay people, the single label “depression” makes the medical diagnosis less relevant.

## Experiment 2

In Experiment 1, although we demonstrated that the effect of the label on the perception that a protagonist has depression is stronger when a medical diagnosis accompanies it than when there is no diagnosis, we surprisingly did not find the main effect of the label. One of the reasons for this result may be the relatively low quality of the measures, as we used only single items when measuring our dependent variables. Second, depressed and never-depressed people may have different illness representations of depression (33). Therefore, in this experiment, we aimed to replicate the results of the previous study, making two significant alterations. First, we asked our participants more detailed questions about the protagonist’s condition and calculated composite scores for the two variables. Second, we invited participants who, in the pre-screening questions, indicated that they experienced depression before or during the moment of the study vs. those who did not experience any depression episodes. We again manipulated the use of the “depression” label and the information about medical diagnosis. We used the same description of Alex as in the previous study. We expected that controlling for the perception of Alex as having a medical condition, (H1) participants in the “depression” label condition would assess the probability that the person described in the vignette has depression as higher than participants in the no-label (description only) condition, and (H2) this effect would be stronger for participants in the medical diagnosis condition compared to those in the no diagnosis condition. Additionally, we expected that (H3) these effects would be moderated by participants’ own experience with depression, such that it would be stronger for participants who experienced depression themselves than for participants who did not experience depression. We preregistered these hypotheses, sample size, exclusion criteria, and analyses at https://aspredicted.org/N2N_B2R. We also analyzed the effect of label, diagnosis, and own experience with depression on the perception of Alex as having a medical condition, controlling for the perception of her having depression for exploratory purposes (this analysis has not been preregistered).

### Materials and methods

We estimated the sample size using an *a prioria priori* power analysis with G*Power (32). Based on the results of our previous study, assuming an interaction effect of η^2^ = .01, an expected power of 1 − β = .80, and a significance level of α = .05, we found that a sample size required to detect such an effect should include 195 per experimental condition. Therefore, we aimed to recruit 195 participants for each of the four experimental conditions in a 2 (label vs. no label) × 2 (diagnosis vs. no diagnosis) × 2 (experience of depression vs. no experience of depression) experimental design, resulting in a total of 1,560 participants. Factoring for potential attrition due to failed attention checks, we recruited *N* = 1,624 U.S. Prolific Academic users to participate in this study in exchange for £0.75. We excluded 98 participants based on their responses to two questions that served as the attention checks, the same as in Experiment 1. The final sample included *N* = 1,526 participants (753 women, 753 men, and 20 with no information) aged 18–93 years (*M* = 42.19, *SD* = 14.01).

This study was conducted simultaneously on two separate groups of participants: participants who were previously or currently experiencing depression (*N* = 766) and participants who had no experience of depression (*N* = 760). After giving informed consent and providing demographic information, participants in each group were assigned to one of four conditions in a 2 (label vs. no label) × 2 (medical diagnosis information vs. no diagnosis information) between-subjects design. Again, participants were asked to read a short description of a person with different final passages depending on the condition, the same as in Experiment 1. The final passage was as follows: in the control condition (*n* = 376), “Alex wondered what was going on with her”; in the “label” condition (*n* = 383), “Alex wondered whether she might have depression”; in the “medical diagnosis” condition (*n* = 387), “Alex went to see a doctor, and the doctor diagnosed that the way she feels is due to the medical condition”; and in the “label + medical diagnosis” condition (*n* = 380), “Alex went to see a doctor, and the doctor diagnosed that the way she feels is due to depression”.

In the next step, participants were asked to answer eight questions, four of which measured the perception of the protagonist as having depression (e.g., “In your opinion, how probable it is that Alex might have depression”, *M* = 78.32, *SD* = 16.41, α = .86), and the remaining four measured the perception of the protagonist as having a medical condition (e.g., “In your opinion, how probable it is that Alex might have some medical condition”, *M* = 70.20, *SD* = 20.83, α = .92), using a scale from 0 = “Very improbable” to 100 = “Very probable” (see preregistration for full list of items). These two sets of questions were averaged to serve as the two dependent variables.

### Analytical approach

We conducted two ANCOVAs in the 2 (label vs. no label) × 2 (diagnosis vs. no diagnosis) × 2 (experience of depression vs. no experience of depression) factorial design. The first ANCOVA (preregistered) examined the perception of Alex as having depression as the dependent variable, with the perception of Alex as having a medical condition as a covariate. The second ANCOVA (not preregistered) examined the perception of Alex as having a medical condition as the dependent variable, with the perception of Alex as having depression as a covariate.

### Results

**Figure 3:**
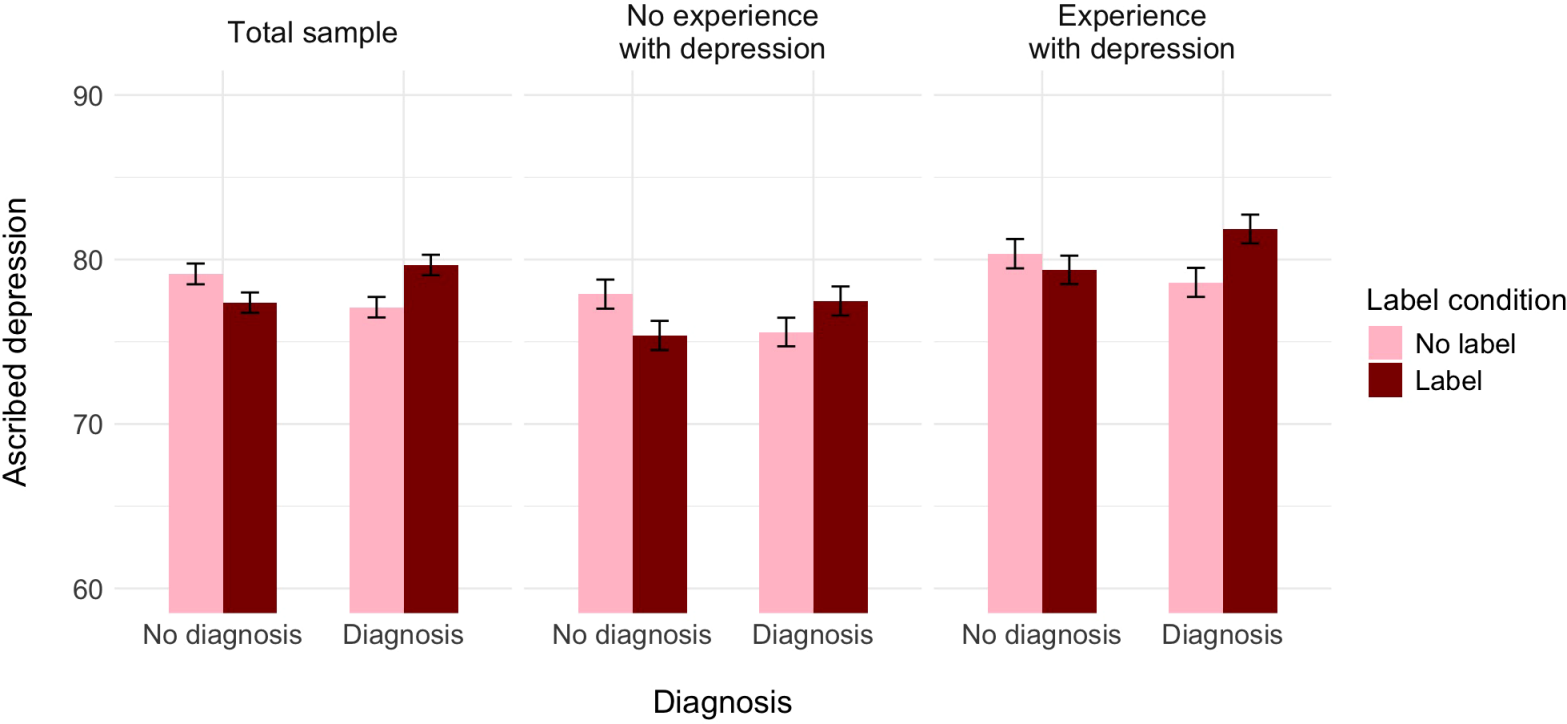
The effect of label, medical diagnosis, and experience with depression on ascribed depression, controlling for the ascribed medical condition (Experiment 2).

In the first analysis, the association between the two dimensions of perception was significant (see **Table 3** and **Figure 3**). The effect of diagnosis was non-significant, the same as the effect of label. The effect of participants’ experience with depression was significant. The only significant interaction was between the two manipulations (diagnosis × label), matching the results of Experiment 1.

**Table 3:** Analysis of covariance for the effect of label, medical diagnosis, and experience with depression on ascribed depression, controlling for ascribed medical condition (Experiment 2).

Predictor	*F* (1, 1,517)	*p*	η^2p^
Ascribed medical condition	1,195.03	<.001	.441
Diagnosis	0.04	.833	.001
Label	0.43	.510	.001
Diagnosis × Label	11.96	<.001	.008
Depression	31.12	<.001	.020
Diagnosis × Depression	0.145	.703	.001
Label × Depression	1.36	.244	.001
Diagnosis × Label × Depression	0.01	.945	.001

A further decomposition of this interaction with the Bonferroni correction revealed that, controlling for the perception of Alex as having a medical condition, participants who did not receive the information about the diagnosis assessed the probability of her having depression as similar independently of whether the label was provided (*M* = 77.38, *SE* = 0.62) or not (*M* = 79.13, *SE* = 0.63), *t*(1517) = 1.99, *p* = .284, Cohen’s *d* = 0.14. In turn, participants who received the information about the diagnosis assessed the probability of Alex as having depression as higher when the label was provided (*M* = 79.67, *SE* = 0.62) than when it was not (*M* = 77.10, *SE* = 1.23), *t*(1517) = −2.57, *p* = .021, Cohen’s *d* = −0.21. Alternative decomposition of the interaction revealed that within the no-label condition, participants assessed the probability of Alex as having depression as similar independently of whether they received the information about the diagnosis or not, *t*(1517) = 2.26, *p* = .144, Cohen’s *d* = 0.17. In turn, within the label condition, participants who did not receive the information about the diagnosis assessed the probability of Alex as having depression as lower than those participants who received the information about both the label and the diagnosis, *t*(1517) = −2.61, *p* = .055, Cohen’s *d* = −0.19.

**Figure 4:**
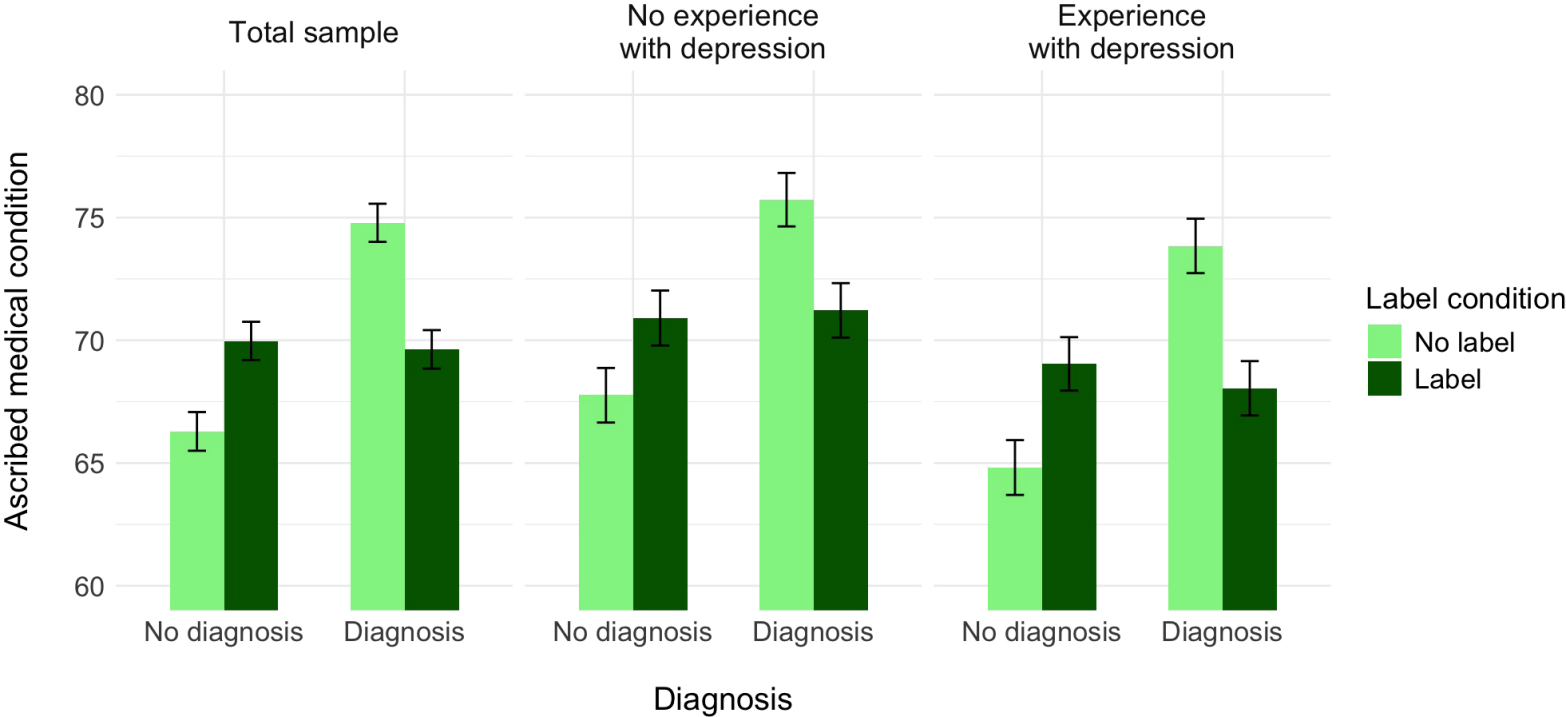
The effect of label, medical diagnosis, and experience with depression on ascribed medical condition, controlling for the ascribed depression (Experiment 2).

In the second analysis, the association between the two dimensions of perception was significant (see **Table 2**). The effect of diagnosis was significant, while the effect of label was insignificant. The effect of participants’ experience with depression was significant, such that those who did not previously experience depression ascribed a higher level of depression (*M* = 71.40, *SE* = 0.56) than those who experienced depression (*M* = 68.94, *SE* = 0.55). The only significant interaction was that between the two manipulations (diagnosis × label), matching the results of Experiment 1 (see **Table 4** and **Figure 4**).

**Table 4:** The effect of label, medical diagnosis, and experience with depression on ascribed medical condition, controlling for ascribed depression (Experiment 2).

Predictor	*F* (1, 1,517)	*p*	η^2p^
Ascribed depression	1,195.03	<.001	.441
Diagnosis	26.84	<.001	.017
Label	0.89	.346	.001
Diagnosis × Label	32.03	<.001	.021
Depression	9.83	.002	.006
Depression × Diagnosis	0.01	.938	.001
Depression × Label	0.01	.947	.001
Depression × Diagnosis × Label	0.57	.450	.001

A further decomposition of this interaction with the Bonferroni correction revealed that, controlling for the perception of the protagonist as having a medical condition, participants who did not receive the information about the diagnosis assessed the probability of Alex as having depression as higher when the label was provided (*M* = 69.97, *SE* = 0.78) than when it was not (*M* = 66.29, *SE* = 0.79), *t*(1517) = −3.33, *p* = .005, Cohen’s *d* = −0.24. In turn, participants who received the information about the diagnosis assessed the probability of Alex as having a medical condition as higher whether the label was not provided (*M* = 74.79, *SE* = 0.78) than when it was provided (*M* = 69.63, *SE* = 0.78), *t*(1517) = 4.68, *p* <.001, Cohen’s *d* = 0.34. Alternative decomposition of the interaction revealed that within the no-label condition, participants who did not receive the information about the diagnosis assessed the probability of Alex as having a medical condition as lower (*M* = 66.29, *SE* = 0.79) than those who received the information about the diagnosis (*M* = 74.79, *SE* = 0.78), *t*(1517) = −7.66, *p* <.001, Cohen’s *d* = −0.56. In contrast, within the label condition, participants who did not receive the information about the diagnosis assessed the probability of Alex as having a medical condition as slightly higher (*M* = 69.97, *SE* = 0.78) than those participants who received the information about both the label and the diagnosis (*M* = 69.63, *SE* = 0.78), *t*(1517) = 0.31, *p* = .999, Cohen’s *d* = 0.02.

In sum, Experiment 2 replicated our findings from Experiment 1. Again, we found that the effect of the label on the perception that a protagonist has depression is significant when a medical diagnosis accompanies it, but not when there is no diagnosis. Furthermore, unlike what we expected, we did not find moderation by participants’ personal experience with depression episodes: the interaction between the label and medical diagnosis seems to be independent of whether participants have such experience.

As in Experiment 1, we additionally found that when a person is diagnosed by a doctor, she/he is perceived as experiencing an actual medical condition to a lower extent when this diagnosis regards depression than when depression is not indicated. Moreover, these effects were also independent of whether the participants had their own experience of depression or not.

## Experiment 3

After demonstrating the effects of label and diagnosis with U.S. participants in Experiments 1 and 2, we conducted a preregistered Experiment 3 to replicate our findings in the UK. The USA and the UK differ substantially in terms of mental health policies and accessibility to mental health care (34): mental health prevention programs are more effective in the UK than in the USA, and also, the accessibility of professional mental health care as well as the public awareness of mental health issues is greater in the UK than in the USA (35, 36). Therefore, replicating our effects of interest in the UK would be a strong robustness check.

Moreover, one shortcoming of these studies may be that the protagonist of the scenario was presented as a woman, and although we used a rather generic name, “Alex”, it is possible that participants may have perceived Alex as a woman. As depression is way more common in women than in men (37) and the gender gap in depression is undisputed (38, 39), this framing may have affected our results. Therefore, in Experiment 3, we explicitly manipulated the gender of the person described in the scenario using names (John vs. Margaret) and gendered pronouns. Finally, although our previous experiments included attention checks, they tested general attention rather than checking whether our participants had read and remembered the scenario about Alex. Therefore, we applied a more specific comprehension check, comprising three questions about the content of the scenario, and we informed our participants that we would pay only those who answered all questions correctly.

In sum, we expected a significant interaction between label depression and information about medical diagnosis such that (H1) when information about medical diagnosis is provided, a protagonist would be perceived as having depression to a greater extent in the label condition than in the no-label condition, but (H2) this effect would be weaker or even insignificant when no information about medical diagnosis is provided. We did not have specific predictions concerning the main effects of diagnosis and label manipulations. We also planned to test whether the abovementioned effect differs across the protagonist’s gender, but we did not have specific expectations concerning the moderating effects of gender. We preregistered these study hypotheses, design, analysis plan, sample size, and data exclusions on https://aspredicted.org/5X2_4LP. We also investigated the effect of label, diagnosis, and protagonist’s gender on the perception of the protagonist as having a medical condition, controlling for the perception of her having depression for exploratory purposes (this analysis has not been preregistered).

### Materials and methods

We estimated the sample size using an *a prioria priori* power analysis with the G*Power software (32), assuming an interaction effect of η^2^ = .01, a power of 1 − β = .80, and α = .05. Based on this analysis, we assumed to have 195 participants for each experimental condition, which is a total number of 1,560 participants in our experimental design. We recruited *N* = 1,594 Prolific Academic users from the UK to participate in this study in exchange for £0.45. We excluded 20 participants based on their responses to comprehension checks. The final sample included *N* = 1,562 participants (775 women, 779 men, and 8 with no information) aged 18–83 years (*M* = 41.88, *SD* = 12.91), and four participants did not provide information about their age.

After giving informed consent and providing demographic information, participants were assigned to one of eight conditions in a 2 (label vs. no label) × 2 (medical diagnosis information vs. no diagnosis information) × 2 (man vs. woman) between-subjects design. In all conditions, they were asked to read a short description of a person (John or Margaret) with different final passages, depending on the condition, as in Experiments 1 and 2. The final passage again differed depending on the experimental conditions.

In the next step, participants were asked to answer the same eight questions that were used to measure our dependent variables in Experiment 2, that is, perception of the John/Margaret as having depression (*M* = 74.63, *SD* = 15.49, α = .81) and as having a medical condition (*M* = 70.10, *SD* = 18.58, α = .90), using a scale from 0 = “Very improbable” to 100 = “Very probable”. Finally, participants were asked to answer three questions on the content of the scenario, with four answers each (only one answer was correct).

### Analytical approach

We conducted two ANCOVAs in the 2 (label vs. no label) × 2 (diagnosis vs. no diagnosis) × 2 (John vs. Margaret) factorial design. The first ANCOVA (preregistered) examined the perception of John/Margaret as having depression as the dependent variable, with the perception of John/Margaret as having a medical condition as a covariate. The second ANCOVA (not preregistered) examined the perception of John/Margaret as having a medical condition as the dependent variable, with the perception of John/Margaret as having depression as a covariate.

### Results

**Figure 5:**
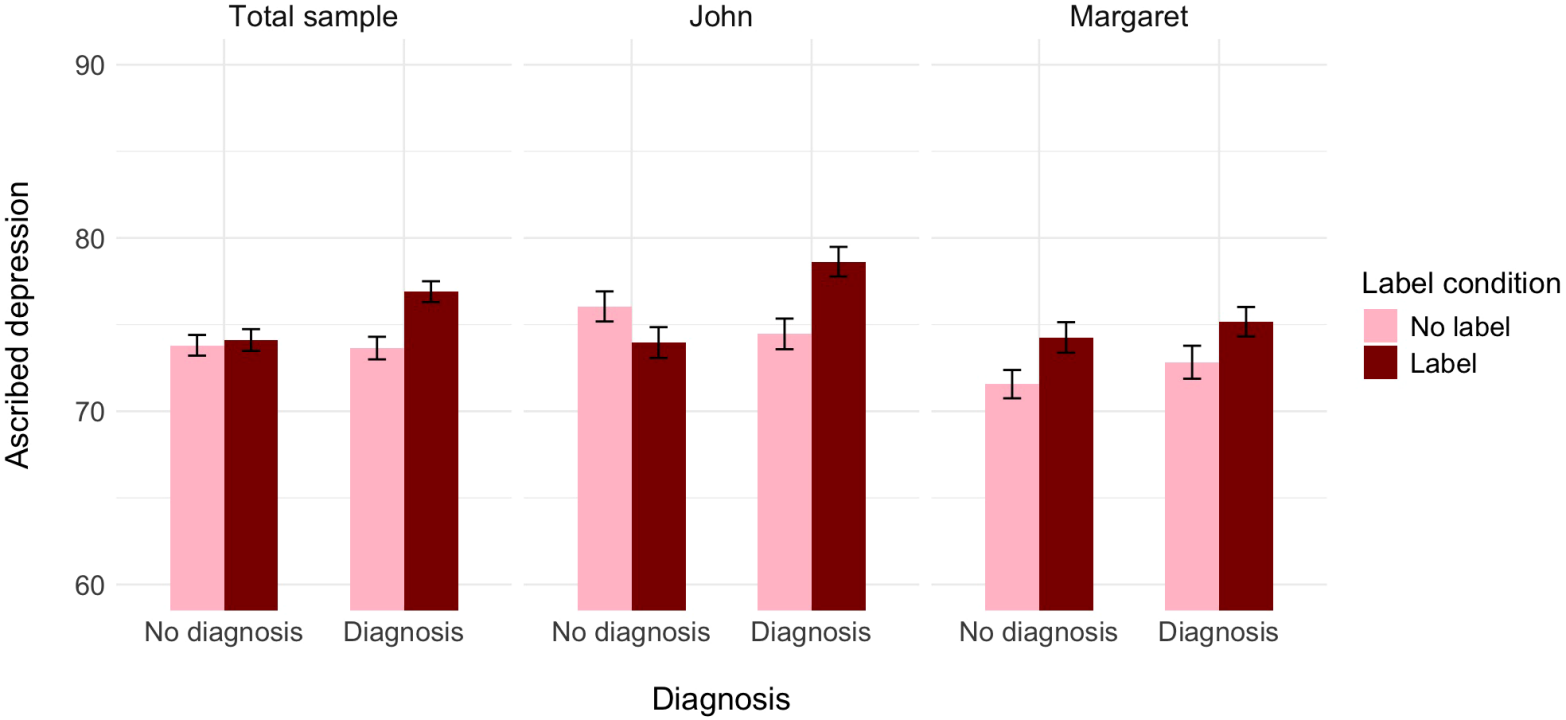
The effect of label, medical diagnosis, and protagonist’s gender on ascribed depression, controlling for the ascribed medical condition (Experiment 3).

In the first analysis, the association between the two dimensions of perception was significant. The main effect of the label was significant, while the main effect of diagnosis was not. The effect of the protagonist’s gender was significant, such that experiencing the same symptoms, John was seen as having depression to a higher extent than Margaret. Most importantly, in line with our preregistered hypothesis, the interaction between information about medical diagnosis and the label was significant. Additionally, we found a significant three-way interaction between label, medical diagnosis, and gender (see **Table 5** and **Figure 5**).

**Table 5:** Analysis of covariance for the effect of label, medical diagnosis, and gender on ascribed depression, controlling for ascribed medical condition (Experiment 3).

Predictor	Total sample			Margaret			John		
	*F* (1, 1,553)	*p*	η^2p^	*F* (1, 778)	*p*	η^2p^	*F* (1, 774)	*p*	η^2p^
Ascribed medical condition	888.13	<.001	.364	489.67	<.001	.386	399.73	<.001	.341
Label	8.30	.004	.005	7.88	.005	.010	1.37	.242	.002
Diagnosis	4.40	.036	.003	0.91	.340	.001	5.13	.024	.007
Label × Diagnosis	5.70	.017	.004	0.01	.948	.001	14.39	<.001	.002
Gender	14.14	<.001	.009						
Gender × Label	1.43	.232	.001						
Gender × Diagnosis	0.14	.713	.001						
Gender × Label × Diagnosis	7.15	.008	.005						

Gender = Protagonist’s gender (John vs. Margaret).

A further decomposition of the two-way interaction between label and diagnosis with the Bonferroni correction again revealed that, controlling for the perception of the protagonist as having a medical condition, participants who did not receive the information about the diagnosis assessed the probability of this person having depression as similar independently of whether the label was provided (*M* = 74.11, *SE* = 0.63) or not (*M* = 73.81, *SE* = 0.60), *t*(1533) = −0.35, *p* = .999, Cohen’s *d* = −0.03. In turn, participants who received the information about the diagnosis assessed the probability of this person as having depression as higher when the label was provided (*M* = 76.90, *SE* = 0.60) than when it was not (*M* = 73.65, *SE* = 0.65), *t*(1533) = −3.68, *p* = .001, Cohen’s *d* = −0.27. Alternative decomposition of the interaction revealed that within the no-label condition, participants assessed the probability of John/Margaret having depression as similar independently of whether they received the information about the diagnosis or not, *t*(1533) = 0.18, *p* = .999, Cohen’s *d* = 0.01. In turn, within the label condition, participants who did not receive the information about the diagnosis assessed the probability of John/Margaret as having depression as lower than those participants who received the information about the label together with the information about the diagnosis, *t*(1533) = −3.20, *p* = .008, Cohen’s *d* = −0.23.

Additionally, we decomposed the three-way interaction that we preregistered for exploratory reasons, investigating whether, controlling for the perception of the protagonist as having a medical condition, the effects of the label and medical diagnosis depend on the protagonist’s gender. Unlike in Experiments 1 and 2, when the person described in the scenario was a woman, we found a significant effect of the label, such that when Margaret used the word “depression”, participants were more prone to see that she has depression (*M* = 74.65, *SE* = 0.65) than when she did not use this word (*M* = 72.10, *SE* = 0.66). The effect of diagnosis was not significant, the same as the interaction between the two manipulations (see **Table 5** and **Figure 5**).

In contrast, when a person in the scenario was described as a man named John, we found a significant interaction between label and diagnosis, accompanied by a significant main effect of diagnosis (see **Table 5** and **Figure 5**). A further decomposition of this interaction with the Bonferroni correction revealed that, controlling for the perception of John as having a medical condition, participants who did not receive the information about the diagnosis assessed the probability of him having depression as similar independently of whether the label was provided (*M* = 74.81, *SE* = 0.86) or not (*M* = 75.53, *SE* = 0.83), *t*(774) = 1.84, *p* = .396, Cohen’s *d* = 0.19. In turn, participants who received the information about the diagnosis assessed the probability of John as having depression as higher when the label was provided (*M* = 78.22, *SE* = 0.84) than when it was not (*M* = 74.60, *SE* = 0.84), *t*(774) = −3.53, *p* = .003, Cohen’s *d* = −0.36. Alternative decomposition of the interaction revealed that within the no-label condition, participants assessed the probability of John as having depression as similar independently of whether they received the information about the diagnosis or not, *t*(774) = 1.03, *p* = .999, Cohen’s *d* = 0.11. In turn, within the label condition, participants who did not receive the information about the diagnosis assessed the probability of John as having depression as lower than those participants who received the information about both the label and the diagnosis, *t*(774) = −4.28, *p* <.001, Cohen’s *d* = −0.44.

**Figure 6:**
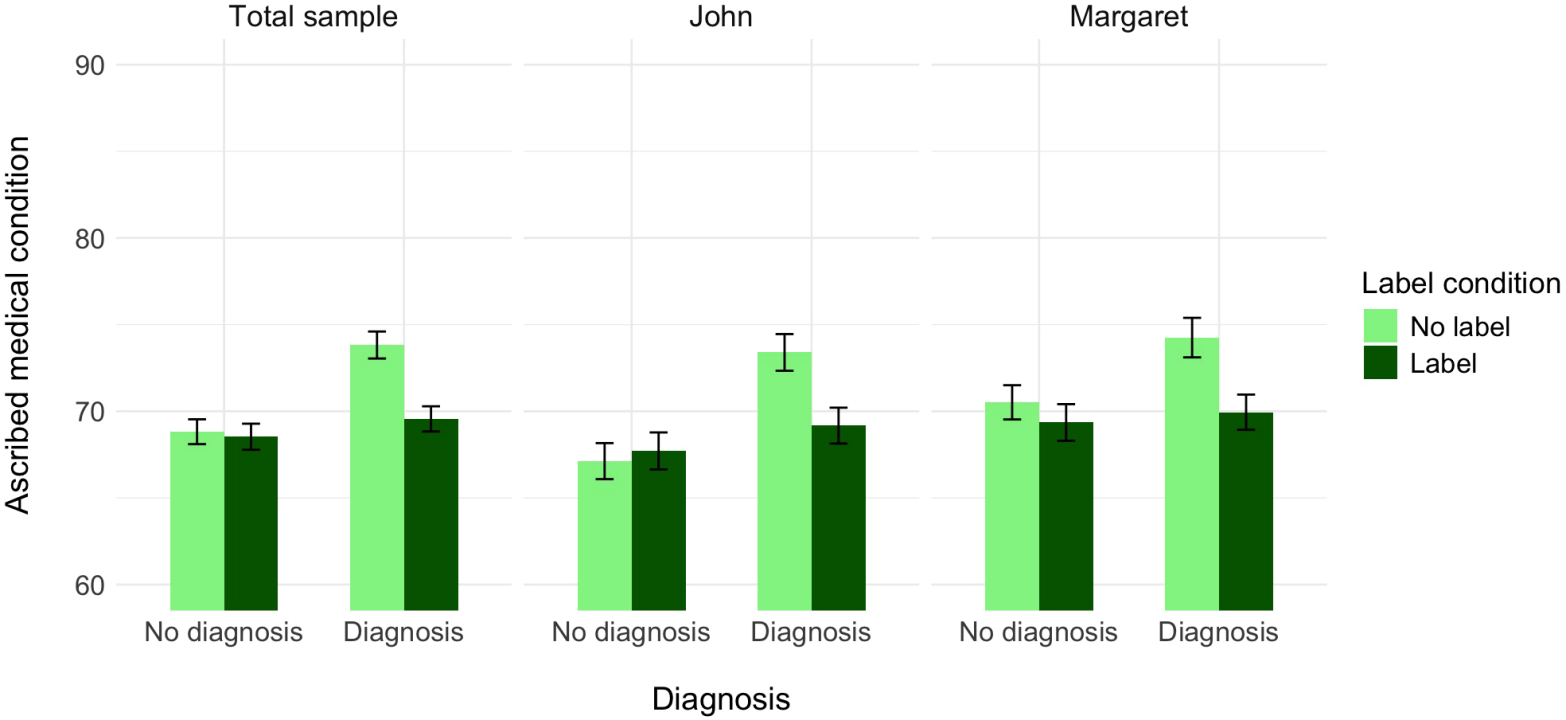
The effect of label, medical diagnosis, and protagonist’s gender on ascribed medical condition, controlling for the ascribed depression (Experiment 3).

In the second analysis, the association between the two dimensions of perception was significant. The main effect of the label was significant, the same as the main effect of diagnosis. The effect of the protagonist’s gender was weak but significant, such that, experiencing the same symptoms, Margaret was seen as having a medical condition to a higher extent than John. Again, the interaction between information about medical diagnosis and the label was significant. None of the two-way interactions with protagonist’s gender and three-way interactions between label, medical diagnosis, and protagonist’s gender were significant (see **Table 6** and **Figure 6**).

**Table 6:** Analysis of covariance for the effect of label, medical diagnosis, and gender on ascribed medical condition, controlling for ascribed depression (Experiment 3).

Predictor	Total sample		
	*F* (1, 1,553)	*p*	η^2p^
Ascribed depression	888.13	<.001	.364
Label	9.40	.002	.006
Diagnosis	16.18	<.001	.010
Label × Diagnosis	7.18	.007	.005
Gender	5.01	.025	.003
Gender × Label	0.38	.537	.001
Gender × Diagnosis	1.31	.252	.001
Gender × Label × Diagnosis	0.31	.575	.001

Gender = Protagonist’s gender (John vs. Margaret).

A further decomposition of the two-way interaction between label and diagnosis with the Bonferroni correction again revealed that, controlling for the perception of the protagonist as having a medical condition, participants who did not receive the information about the diagnosis assessed the probability of this person having a medical condition as similar independently of whether the label was provided (*M* = 68.82, *SE* = 0.72) or not (*M* = 68.53, *SE* = 0.75), *t*(1533) = −0.27, *p* = .999, Cohen’s *d* = −0.02. In turn, participants who received the information about the diagnosis assessed the probability of this person as having a medical condition as lower when the label was provided (*M* = 69.56, *SE* = 0.73) than when it was not (*M* = 73.82, *SE* = 0.78), *t*(1533) = 4.01, *p* <.001, Cohen’s *d* = 0.29. Alternative decomposition of the interaction revealed that within the no-label condition, participants assessed the probability of John/Margaret having a medical condition as higher when they received the information about the diagnosis vs. when they did not have such information, *t*(1533) = −4.72, *p* <.001, Cohen’s *d* = −0.34. In turn, within the label condition, participants assessed the probability of John/Margaret as having a medical condition as similar irrelevant of whether they received the information about the label together with the information about the diagnosis or not, *t*(1533) = −0.98, *p* = .999, Cohen’s *d* = −0.07.

To summarize, for the perception of the protagonist as having depression, the results of Experiment 3 for the male protagonist replicate our results of Experiments 1 and 2. Again, while we did not find a significant main effect of label, we did find that the effect of label on the perceived probability that a protagonist has depression was significant when a medical diagnosis was present, but not when no diagnosis was present. For the female protagonist, we found an effect of label independent of the effect of medical diagnosis on the perception that the protagonist has depression.

For the perception that the protagonist has a medical condition, the results replicated our findings from Experiments 1 and 2 for both protagonists. The label “depression” consistently decreased perceived medical legitimacy when a diagnosis was present, and this pattern was identical for both John and Margaret. Unlike the first dependent variable (perception of having depression), where gender moderated the effect of labeling, the effect of labeling on perceived medical legitimacy remained consistent regardless of the protagonist’s gender. These results indicate that while gender has an effect on how depression labels interact with medical diagnoses in the perceived attribution of depression, it has no effect on how these labels influence the perceived legitimacy of the medical condition itself.

## Meta-analysis

To strengthen our claim that using the label “depression” triggers the perception of a person as having depression, but only if it is accompanied by a medical diagnosis, we performed a meta-analysis on Experiments 1–3. To account for the variance caused by different study designs and participant populations, we performed random-effects meta-analyses using the Multicondition Meta-Analysis (MCDM) software dedicated to single-paper meta-analyses (40). Given our main hypothesis that the label “depression” triggers the perception of a person as having depression, but only if a medical diagnosis accompanies it, we were primarily interested in the label × diagnosis interaction effects in our meta-analysis. We also investigated the main effects of both manipulations, as well as the simple effect of the label when participants were informed about the diagnosis or not.

### Methods

For Experiment 1, we defined and coded four subgroups, depending on whether the protagonist used the label “depression” and whether information about medical diagnosis was included in the scenario. As Experiment 2 included participants who had a history of depression and those who did not, we decided to code it as two different studies in a 2 (no label vs. label) × 2 (no diagnosis vs. diagnosis) design. Finally, Experiment 3 included a scenario of a male protagonist (John) and a female protagonist (Margaret). Hence, we also coded it as two separate studies in the same 2 × 2 design.

### Results

The results of the analysis revealed a marginally significant main effect of the label, *b* = 0.17, *se* = 0.09, *Z* = 1.87, *p* = .061, a significant main effect of medical diagnosis, *b* = 0.20, *se* = 0.09, *Z* = 2.16, *p* = .031, and a reliable significant interaction between the two manipulations, *b* = −0.35, *se* = 0.09, *Z* = −3.78, *p* <.001. Further analyses confirmed the effect of label in the presence of diagnosis, *b* = 0.26, *se* = 0.07, *Z* = 3.98, *p* <.001, but a lack of such effect in the absence of diagnosis, *b* = −0.09, *se* = 0.05, *Z* = −1.35, *p* = .177. In sum, the meta-analysis supports our hypothesis that medical diagnosis legitimizes participants’ attribution of depression to the protagonist, as across our three experiments, participants ascribed a higher level of depression to a protagonist when depression was diagnosed by the doctor, compared to all other conditions.

Furthermore, we analyzed *I* ^2^, a statistical measure that describes the percentage of variation in the observations (beyond that attributable to the experimental manipulations) due to heterogeneity (40). In our case, we estimated *I* ^2^ at 49.62%, *Q*(12) = 23.82, *p* = .022, suggesting that method factors account for approximately half of the variation in the observations beyond that attributable to experimental manipulations. According to guidelines on the typical *I* ^2^ size in behavioral research (41), an *I* ^2^ of approximately 50% indicates medium heterogeneity. However, the uncertainty interval for our *I* ^2^ was 95% CI [4.65%, 73.38%], suggesting that the data are consistent with there being anywhere from low to high heterogeneity, and the estimate of heterogeneity is imprecise. For that reason, we also tested whether participants’ nationality, a factor varying across our experiments, impacted the effect of label × diagnosis interaction on ascribed depression. An additional test yielded no effect of the country on the interaction, *b* = 0.18, *se* = 0.19, *Z* = 0.94, *p* = .350, on the main effect of label, *b* = 0.21, *se* = 0.19, *Z* = −1.07, *p* = .278, and on the main effect of diagnosis, *b* = 0.03 *se* = 0.19, *Z* = 0.16, *p* = .869. After controlling for this study-level moderator, the general interaction effect remained significant, *b* = −0.60, *se* = 0.29, *Z* = −2.09, *p* = .037. Moreover, the estimate of heterogeneity slightly increased rather than decreased, *I* ^2^ = 53.02%, 95% CI [3.82%, 77.05%], *Q*(9) = 19.16, *p* = .024, suggesting that the majority of unexplained variance was due to various factors other than country.

Additionally, we conducted a similar analysis for the perception of the protagonist as having a medical condition. The results of the analysis revealed a significant main effect of the label, *b* = −0.27, *se* = 0.07, *Z* = −3.73, *p* <.001, a significant main effect of medical diagnosis, *b* = 0.50, *se* = 0.07, *Z* = 6.86, *p* <.001, and a significant interaction between the two manipulations, *b =* 0.43, *se* = 0.07, *Z* = 5.83, *p* <.001. Further analyses confirmed the effect of diagnosis in the absence of label, *b* = 0.47, *se* = 0.05, *Z* = 8.96, *p* <.001, but a lack of such effect in the presence of label, *b* = 0.04, *se* = 0.05, *Z* = 0.73, *p* = .463. In sum, the meta-analysis strongly supported our hypothesis that using the label “depression” for self-description somehow “nullifies” the effect of medical diagnosis, as across our studies, participants ascribed a higher level of disorder to a person who had a diagnosis formulated by the doctor, but only when this diagnosis did not refer to depression. In other words, even if the symptoms described in the scenario are the same across four conditions, they are not seen as indicating a medical condition if depression is mentioned.

Again, we analyzed *I* ^2^ for this analysis and found that it was much lower than for the previous analysis. In our case, we estimated *I* ^2^ at 20.77%, *Q*(12) = 15.15, *p* = .234, 95% CI [0%, 58.41%], suggesting that method factors account for approximately a quarter of the variation in the observations beyond that attributable to experimental manipulations, which indicates relatively low heterogeneity. We again tested whether participants’ nationality impacted the effect of label × diagnosis interaction on ascribed depression. An additional test yielded a significant effect of the country on the interaction, *b* = −0.28, *se* = 0.13, *Z* = −2.16, *p* = .031, while there was no moderation effect of the country on the label, *b* = −0.11, *se* = 0.13, *Z* = −0.84, *p* = .400, and on the diagnosis, *b* = −0.14, *se* = 0.13, *Z* = −1.06, *p* = .289. After controlling for this study-level moderator, the focal interaction effect remained significant, *b* = 0.40, *se* = 0.07, *Z* = 4.20, *p* <.001. At the same time, the estimate of heterogeneity decreased, *I* ^2^ = 0%, 95% CI [0%, 60.52%], *Q*(9) = 8.58, *p* = .477, suggesting that some unexplained variance was due to country. In conclusion, the focal interaction was stronger in the USA than in the UK.

## General discussion

In a series of three experiments, we investigated whether the perception of a person experiencing symptoms of depression as actually experiencing depression and as actually experiencing a medical condition is affected by the use of the label “depression” and the additional information that this condition was diagnosed by a doctor. We demonstrated the following: 1) participants perceived the protagonist as actually having depression to a greater extent when the label was accompanied by information that the depression had been diagnosed by a doctor, but not when there was no information about the diagnosis, and that the use of the label “depression” reduced the perception of the protagonist as experiencing an actual medical condition, even when the disorder was diagnosed by a doctor. 2) These effects were not moderated by participants’ own experience of depression. 3) We replicated the aforementioned results but only for the male protagonist; for the female protagonist, we found that the effect of the label was independent of the effect of the medical diagnosis. Finally, the results of a meta-analysis on Experiments 1–3 supported our hypothesis that the use of the label “depression” weakened the effect of the medical diagnosis, as participants attributed a higher probability of an actual medical condition to a person who had a diagnosis formulated by a doctor, but only when this diagnosis did not include the label “depression”.

Previous research on the effects of labeling has focused on attitudes and beliefs about people diagnosed with various mental disorders: their perceived competence, dangerousness, etc. To our knowledge, this project is the first to aim at a systematic investigation of the labeling effect on public perceptions of the experience of depressive symptoms as medically based and justified, rather than on the perceptions of a person diagnosed with depression. Our findings both align with and extend prior research on diagnostic labeling effects. Consistent with studies showing that psychiatric labels can influence perceptions of disorder (42), we found that the “depression” label altered perceived medical legitimacy. Moreover, our results help explain inconsistencies in research on medical explanations for mental disorders. While medical or biogenetic explanations may reduce blame and promote help-seeking (43), they do not consistently increase overall credibility of mental disorders and may even increase stigmatization, social distance, and pessimism about recovery. Our results show that the credibility of medical explanations is only increased when the diagnosis avoids the specific term “depression”.

These findings may reflect the dual nature of “depression” in public discourse, functioning both as a clinical term and as everyday language for sadness or low mood. When the public encounters the label “depression”, they may activate associations with common emotional experiences rather than severe psychiatric conditions, thereby reducing perceived medical legitimacy. This interpretation aligns with research on semantic networks and concept accessibility (44), where familiar terms may prime non-medical associations that compete with clinical interpretations. These results suggest a need to clarify what “depression” means from a medical perspective and how it differs from everyday experience. This seems particularly important given that the illness representation of depression as constructed by people who actually experience it differs markedly from the representation shared by their caregivers (45) as well as mental health professionals (46–48). In sum, the way depression is portrayed and described may affect self-stigma, treatment effectiveness, and beliefs about depression (49).

Although the effects we found are rather small, we believe that identifying any smaller effects is crucial for understanding what the term “depression” actually means to the public and how lay people perceive the legitimacy of the condition. The number of people diagnosed with depression is systematically and steadily increasing, but the number of people seeking professional help is not growing accordingly. Hence, we believe that even these small effects could be beneficial for public health. Our results could also be useful in communicating diagnoses: as we demonstrated that using the term “depression” reduces the perceived legitimacy of the disorder, clinicians may consider how they communicate a diagnosis of depressive disorder to patients and their families to avoid misunderstandings. For example, clinicians may benefit from using more specific clinical terminology (e.g., “major depressive disorder” or “clinical depression”) or providing additional context that distinguishes the medical condition from everyday sadness. This approach could enhance treatment engagement and reduce the risk of patients or families minimizing the seriousness of the diagnosis. As effective communication impacts further help-seeking, treatment outcomes, and satisfaction of mental health service users (50–52), the importance of comprehensive communication between service users and clinicians seems crucial. From a public health perspective, these findings suggest that mental health literacy campaigns should address the distinction between clinical depression and everyday emotional experiences, potentially reducing misconceptions that may delay help-seeking behaviors.

An important secondary finding concerns the role of participants’ personal depression experience. Although this was not our primary focus, we found a significant effect of participants’ own experience with depression in Experiment 2. This suggests that such experience influences how individuals interpret depression-related information in others, which has potential clinical relevance. Individuals with personal depression experience may have different frameworks for understanding depression legitimacy, possibly due to their firsthand knowledge of symptom severity or treatment experiences. This finding aligns with research showing that mental health literacy varies significantly between those with and without personal experience of mental illness (53). Future research should systematically examine how personal depression experience shapes perceptions of depression in others, as this could inform targeted educational interventions.

Another important result that warrants further investigation comes from Experiment 3, in which we manipulated the gender of the protagonist. While the male protagonist showed results consistent with those of our female protagonists from Experiments 1 and 2, the female protagonist in Experiment 3 showed a different pattern, with label effects independent of medical diagnosis. This suggests possible gender differences in the way depression labels and medical diagnoses interact in public perception. However, as gender was only manipulated in one experiment, systematic replication across multiple studies would be necessary to establish the reliability and generalizability of this effect before definitive conclusions can be drawn about gender differences in perceptions of depression.

Future research should also systematically investigate the boundary conditions of these labeling and diagnosis effects, particularly examining individual differences and cross-cultural variations that may moderate public perceptions of depression. Understanding when and for whom these effects occur is crucial for developing targeted interventions and communication strategies. In our studies, we were unable to identify individual differences that moderate the effect of labeling and diagnosis, having examined only one potential moderator—participants’ personal experience with depression. Other promising candidates for moderation effects include beliefs about depression, such as depression literacy, defined as the knowledge about depression derived from evidence-based and scientific facts about this disorder, and depression misconceptions, understood as the culturally and socially shaped false knowledge about depression (54). Similarly, we see a need for cross-cultural research to understand how different cultural contexts shape the relationship between depression labeling, medical diagnosis, and perceived legitimacy. These individual difference factors and cultural variations can influence not only the perception of depression as an actual health disorder but also the perceived severity of this disorder. Potential benefits of this area of study include better-tailored interventions for anti-stigma campaigns and reshaping the narrative about depression. This could lead to the development of public health messages that address both depression literacy and misconceptions about depression, which in turn can improve public understanding of mental health.

Whereas this research provides a systematic investigation on how the “depression” label and the information about a medical diagnosis shape lay perceptions of people with depression as legitimately experiencing depression and a medical condition, it is important to acknowledge its limitations. One limitation is that Experiment 1 relied on a single question that served as the measure of the dependent variables. We addressed this issue in Experiments 2-3 using more detailed, multi-item composite scores. We also addressed a possible limitation of Experiments 1 and 2– regarding the gender-neutral name “Alex” that could have been perceived as a female or male name and potentially influenced our results, given that depression is more commonly diagnosed in women. Although Experiment 3 directly addressed this by manipulating the protagonist’s gender, the divergent findings—where the effect of the label was independent of diagnosis for the female protagonist but not for the male—highlight complexities that need further investigation.

This research project as a whole has additional constraints. First, the studies were based on written vignettes, which may not capture the full complexity of real-world public perceptions or cultural differences in understanding the phenomenon of depression. Second, although the results were consistent across studies and participants’ countries of origin, the observed effect sizes were small. Third, we did not explore the underlying mechanisms for these effects or address how labeling may impact the perceived severity of depression. Fourth, our samples were predominantly from Western, educated populations, limiting generalizability to other cultural contexts where concepts of mental illness, medical authority, and stigma may differ substantially. Finally, our vignette methodology, while providing experimental control, cannot capture the dynamic nature of real-world interactions where contextual factors, non-verbal cues, and interpersonal relationships may significantly influence how depression labels and diagnoses are perceived and processed.

## Data Availability

The datasets presented in this study can be found in online repositories. The names of the repository/repositories and accession number(s) can be found below: https://researchbox.org/3167.
